# A phosphate glass reinforced composite acrylamide gradient scaffold for osteochondral interface regeneration

**DOI:** 10.1016/j.bbiosy.2024.100099

**Published:** 2024-07-26

**Authors:** Zaid M. Younus, Ifty Ahmed, Paul Roach, Nicholas R. Forsyth

**Affiliations:** a School of Pharmacy and Bioengineering, Keele University, Keele, UK; b College of Pharmacy, University of Mosul, Mosul, Iraq; c Faculty of Engineering, Advanced Materials Research Group, University of Nottingham, Nottingham, UK; d Department of Chemistry, School of Science, Loughborough University, Leicestershire, UK; e Vice principals’ office, King's College, University of Aberdeen, Aberdeen, AB24 3FX, UK

**Keywords:** Osteochondral, Phosphate glass, Gradient, Chondrogenic, Osteogenic, Mineralization

## Abstract

The bone-cartilage interface is defined by a unique arrangement of cells and tissue matrix. Injury to the interface can contribute to the development of arthritic joint disease. Attempts to repair osteochondral damage through clinical trials have generated mixed outcomes. Tissue engineering offers the potential of integrated scaffold design with multiregional architecture to assist in tissue regeneration, such as the bone-cartilage interface. Challenges remain in joining distinct materials in a single scaffold mass while maintaining integrity and avoiding delamination. The aim of the current work is to examine the possibility of joining two closely related acrylamide derivatives such as, poly n-isopropyl acrylamide (pNIPAM) and poly n‑tert‑butyl acrylamide (pNTBAM). The target is to produce a single scaffold unit with distinct architectural regions in the favour of regenerating the osteochondral interface. Longitudinal phosphate glass fibres (PGFs) with the formula 50P_2_O_5_.30CaO.20Na_2_O were incorporated to provide additional bioactivity by degradation to release ions such as calcium and phosphate which are considered valuable to assist the mineralization process. Polymers were prepared via atom transfer radical polymerization (ATRP) and solutions cast to ensure the integration of polymers chains. Scaffold was characterized using scanning electron microscope (SEM) and Fourier transform infra-red (FTIR) techniques. The PGF mass degradation pattern was inspected using micro computed tomography (µCT). Biological assessment of primary human osteoblasts (hOBs) and primary human chondrocytes (hCHs) upon scaffolds was performed using alizarin red and colorimetric calcium assay for mineralization assessment; alcian blue staining and dimethyl-methylene blue (DMMB) assay for glycosaminoglycans (GAGs); immunostaining and enzyme-linked immunosorbent assay (ELISA) to detect functional proteins expression by cells such as collagen I, II, and annexin A2. FTIR analysis revealed an intact unit with gradual transformation from pNIPAM to pNTBAM. SEM images showed three distinct architectural regions with mean pore diameter of 54.5 µm (pNIPAM), 16.5 µm (pNTBAM) and 118 µm at the mixed interface. Osteogenic and mineralization potential by cells was observed upon the entire scaffold's regions. Chondrogenic activity was relevant on the pNTBAM side of the scaffold only with minimal evidence in the pNIPAM region. PGFs increased mineralization potential of both hOBs and hCHs, evidenced by elevated collagens I, X, and annexin A2 with reduction of collagen II in PGFs scaffolds. In conclusion, pNIPAM and pNTBAM integration created a multiregional scaffold with distinct architectural regions. Differential chondrogenic, osteogenic, and mineralized cell performance, in addition to the impact of PGF, suggests a potential role for phosphate glass-incorporated, acrylamide-derivative scaffolds in osteochondral interface regeneration.

## Highlights

• Combining two acrylamide hydrogels, poly n-isopropyl acrylamide (pNIPAM) and poly n‑tert‑butyl acrylamide (pNTBAM), created multi-regional scaffold featuring 3 different zones with different porosities.
• The different scaffold's zones supported osteogenic and chondrogenic cells differentiation with more chondrogenic performance by poly N‑tert-butylacrylamide (pNTBAM).
• The middle scaffold's zone addressed for more mineralization potential by chondrocytes and osteoblasts.
• The phosphate glass fibres incorporated across scaffold's length, enhanced osteogenic and mineralization performance by cells.
• The produced multi-regional scaffold may support the regeneration of the complex multi-zonal osteochondral interface.

## 1 Introduction

The osteochondral region has a unique structural composition ranging from mineralised sub-chondral bone to a more flexible cartilage region [1]. Within this region, a gradual transformation creates a tissue gradient that supports the functional integrity and flexibility of joints [2]. Osteochondral defects cause impairment to the smooth, gliding motion of the joints, which cause discomfort, edema, stiffness, and a decline in joint function [3,4]. These defects can further deteriorate over time if left untreated, potentially leading to osteoarthritis, joint degeneration, and long term disability [5,6]. Treatment strategies, rely primarily on stimulation of inflammatory responses and cellular regeneration of damaged tissues, have limitations in their lack of specificity of outcome tissue type and requirement for multiple surgical interventions [[7], [8], [9]].

The tissue engineering dogma describes an extracellular matrix (ECM) substitute that will act as a template to support cell growth and differentiation. Conceptually, this creates a three-dimensional environment that matches the natural ECM and favours cell adhesion, proliferation, and differentiation [10]. A wide range of materials with tissue-specific features may support this goal by promoting cell specific functions to regenerate damaged tissues [[11], [12], [13]]. The specific chemistry and surface features of materials may significantly alter cell proliferation and attachment, thus may serve the purpose of regenerating a complex tissue construct[14]. Moreover, fabrication of polymers into hydrogel scaffolds can offer more cell adaptive properties[15]. The complex multi-scale structure of the osteochondral region may thus require a combination of two or more biomaterials in order to mimic the complex natural tissue. We searched the literatures through PubMed, Science direct, and Google Scholar for the last 10 years utilizing key words such as multi-material scaffold design, and gradient scaffolds for osteochondral tissue regeneration. We also searched for bioactive materials and bioactive glass re-enforced scaffolds for osteochondral tissue regeneration within the same period. Studies have shown some progress by joining materials together to create a multi (bi- or tri-phasic) scaffold to guide the regeneration of certain tissues including the osteochondral region[16]. However, there are challenges in combining these materials together into one unit; many have used protein glue to attach scaffold layers producing an integral multi-layered construct [17,18]. Irrespective, problems from de-mixing and delamination create more challenges in multi-layered scaffold production [19,20]. Additional strategies to regenerate osteochondral tissues composed of gradient scaffolds utilizing specific techniques such as freeze-drying, solvent casting, electrospinning, and 3D printing, have yielded promising results according to in vitro and in vivo experimental models [[21], [22], [23]]. However, some drawbacks have arisen in terms of the consistency, mechanical, and type of tissue regenerated within long term follow-up [24]. Other techniques that rely mostly on bioreactors such as shear forces, and physical stimuli can be time consuming and costly [25].

Alongside multi-materials combinations, the addition of bioactive substances, such as growth factors or adhesive peptides, to the hydrogel structure may also enhance cell signalling causing increased spreading and proliferation[26,27]. Moreover, some bioactive ions, such as those released from bio-active resorbable glasses, may encourage cells’ osteoblastic and chondrogenic performance and has thus shown positive results when incorporated within osteochondral scaffolds [28]. Our target was to focus on materials’ chemistry to generate a scaffold unit through single polymerization reaction and to rely on polymers crosslinking to bond polymers’ regions.

In an attempt to create a multi-regional scaffold while avoiding the problems of delamination or de-mixing, we searched materials with different chemistry and characteristics but belong to same family such as the acrylamide polymers. Acrylamide based polymers have been widely explored for the purpose of tissue regeneration due to their versatility in pore generation and fabrication, and biomimetic characteristics [29,30]. Polyacrylamide hydrogels have an exceptional capacity for water absorption and retention, which makes them extremely biocompatible as they closely mimic the extracellular matrix of tissues [31]. Additionally, these hydrogels possess tuneable mechanical characteristics that enable them to match the stiffness of a range of tissues and offer suitable support for cellular development and differentiation. The internal porous architecture of these hydrogels also allows nutrients, and waste compounds to diffuse more easily, enhancing cell survival and tissue ingrowth [29,32,33]. In light of this, we sought to make use of the variable chemistry and tuneable properties of acrylamide hydrogels to establish a scaffold unit capable of addressing the regeneration of the osteochondral interface. Building on our previous report we selected poly N-isopropylacrylamide (pNIPAM) and poly N-*tert*-butylacrylamide (pNTBAM) for further development supported further by their applications in tissue engineering and drug delivery systems [34]. Additional methyl modification to NIPAM gives rise to NTBAM [35,36] with more hydrophobic characteristic, [37] affecting phase separation tendency and therefore pore structure during fabrication of pNTBAM gels. Tengvall, et al. noted that materials presenting CH3 groups can bind proteins more efficiently than those with OH groups [38]. The variable number of CH3 groups marking NIPAM and NTBAM polymers may thus offer strong biocompatibility with variable cell responses. Moreover, scaffolds fabrication by combining these polymers together or with other polymer has been highlighted to have a favourable impact in biomedical applications [39,40]. As such, these polymers may be convenient for creating a single unit scaffold with variable surface features. These variable features, mostly in terms of wettability and stiffness, may variably support osteogenic to chondrogenic differentiation and thus our current goal of building osteochondral scaffold.

In the current work, we aimed to utilise pNIPAM and pNTBAM to create a single hydrogel scaffold that ensured the engagement of both polymers into one unit. We hypothesized that the process of scaffold production along with polymer elongation and cross linking could provide the bonding step driving their combination. We further hypothesized that the resultant scaffold would likely consist of 3 variable compartments starting from one polymer through the mixed interface and reaching to the other polymer zone. Using this bi-phasic polymer system a gradient architecture could be generated to mimic the physical structure of the osteochondral interface.

As part of our plan to support the regeneration of the osteochondral interface, we sought to incorporate Phosphate-glass fibres (PGFs) into the pNIPAM-pNTBAM scaffold. PGFs are fully resorbable osteoconductive materials composed mainly from calcium and phosphate (basic formula 50P2O5.30CaO.20Na2O) [41] that have shown to support mineralization and encourage osteogenic behaviour of cells [[42], [43], [44]]. The phosphate-based glass fibres mainly contain calcium and phosphate as the main constituents, both being essential for bone mineralization, and can be control released as the PGFs degrade [45,46]. We hypothesized that the PGFs would aid the design of the current scaffold in two ways; they would leave empty channels after degradation allowing for cells migration; and they will potentially promote mineralization (via release of calcium and phosphate ions) enhancing osteogenic differentiation and function through promotion of calcification of chondrocytes.

## 2 Materials and methods

### 2.1 Materials and sources

Details of materials utilized in the current study were mentioned accordingly within the following steps of methodology. A list of essential materials and kits are provided as a supplementary material with the manuscript.

### 2.2 Preparing individual monomeric solutions

Polymerization of NIPAM and NTBAM monomers into a hydrogel network was mediated by the process of atom transfer radical polymerization (ATRP) [47]. Briefly, NIPAM (4% w/v) was dissolved in 1 mL of deionized water, NTBAM (7.9% w/v) was dissolved in 1 mL of water: methanol (99 %) mixture (1:1 v/v solvent mixture). A 0.1% w/v of cross linker N,N′-methylenebisacrylamide (MBA) was used to link the polymer network by forming covalent bonding between growing polymer chains. The MBA cross linker was added to the monomeric solutions before the initiation of polymerization process. Solutions were then purged with nitrogen gas for 10–15 min, through an 18-gauge needle, at a rate of approximately 10–15 bubbles/second, to evacuate oxygen from the solution. The rate of bubbling was regulated by a pressure control valve adjusted at 2 pounds per square inch gauge (psig) at room temperature.

### 2.3 Gradient scaffold manufacture

**Fig. 1:**
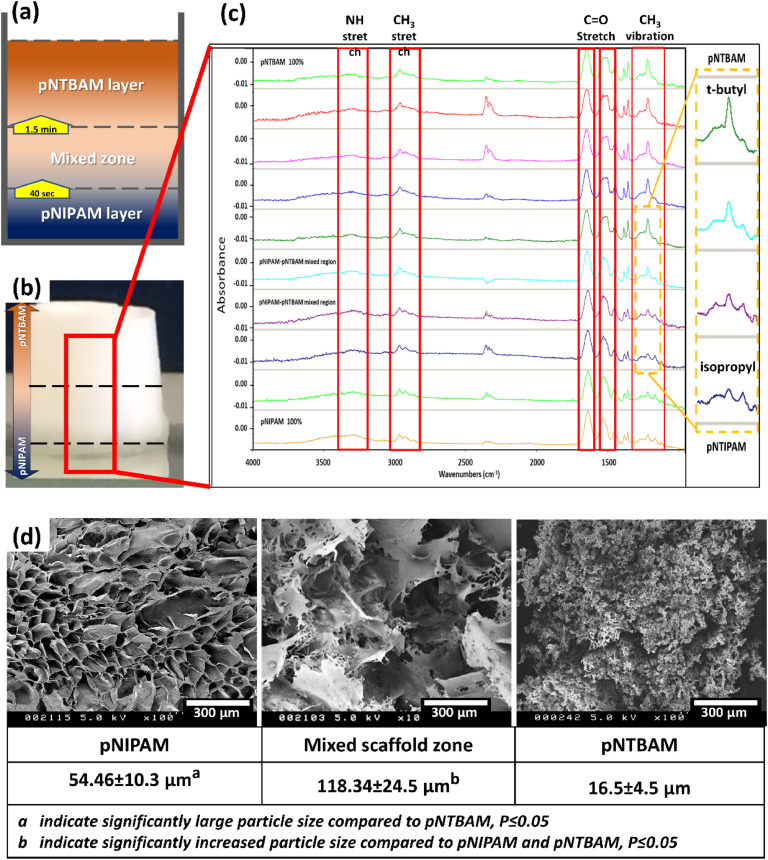
Generation, physical appearance, and characterisation of gradient hydrogel scaffold. (a) Cast scaffold with pNIPAM layer at the bottom and pNTBAM layer on top, yellow arrows indicating time duration before addition of the next polymer; (b) General scaffold appearance, arrows indicate the range of each polymer region; (c) FTIR spectra across the length of gradient scaffold working from pNTBAM layer through to pNIPAM layer. Red rectangles correspond to the main peaks identified in pNIPAM and pNTBAM FTIR spectra. The peak outlined at the low energy region corresponds to the fingerprint region specific for each polymer. The elevated peak from pNTBAM gradually transformed into two small peaks for pNIPAM. The yellow dotted marked region represents the mixed polymer regions and peak transformation. (d) SEM imaging of the three regions of the resultant scaffold featuring pNIPAM, mixed scaffold zone, and pNTBAM with the corresponding mean pore diameter ±SD.

NIPAM and NTBAM polymers were cast in a 24-well plate; the monomeric solutions were added sequentially based on polymerization times for each. pNIPAM formation takes about 2–3 min for full polymerization to complete while pNTBAM polymerization required up to 10–15 min. Creating multi-material scaffolds depends on casting materials layer by layer using variable techniques to obtain a 3D construct [48,49]. Our strategy is adapted from these previous works with integration of timing of polymer addition and initiation of polymerisation. As the purpose of the present work is to produce a single scaffold mass with chemically integrated multilayers, it was technically relevant that the sequential mixing be performed while the polymerization process in progress. This will allow polymer layers to infiltrate each other at their interfaces forming a stable bonding. Immediately before addition, each monomeric solution was mixed with 15 µL of 10% w/v ammonium persulfate (APS) to initiate the polymerization reaction. NIPAM solution (0.5 mL) was added to the container and after 40 s (NIPAM polymerisation initiation) pNIPAM-pNTBAM (0.5 mL) mixture was added followed by adding 0.5 mL NTBAM solution exactly 1 min later due to polymerisation times of each component (Fig. 1a). The timing enabled creation of an integrated co-polymerised scaffold construct. Samples were sealed and left overnight at room temperature for polymerisation to run to completion.

### 2.4 Characterisation of gradient scaffold

#### 2.4.1 Fourier transform infra-red (FTIR) spectroscopy

Scaffold samples were frozen at −20 °C for 24 h followed by 24 h freeze-drying (pressure of 1 torr at −20 °C) using Edwards freeze dryer machine to minimize water noise in spectra. Dried scaffolds were then dissected into vertical and horizontal sections. FTIR analysis was performed to map across the gradient composite regions using a ThermoScientific IS50 FTIR fitted with a single bounce germanium ATR. Data were recorded in Omnic at 4 cm⁻1 resolution between 4000 and 400 cm-1, with 32 scans averaged for each spectra recorded.

#### 2.4.2 Scanning electron microscopy (SEM)

Freeze-dried scaffolds were sliced into small multiple sized pieces (1–2 mm3) and mounted over a carbon plate covered metal holder, gold coated, and viewed by SEM at 5 kV. Observations were recorded using a bench top Hitachi S4500 (SEM). A minimum of 3 specimens from each scaffold region were examined (pNIPAM, pNTBAM, and the mixed interface). Images, scaled at 300 µm, were processed in Image J to measure pore diameter using the straight-line measurement tool [50,51].

### 2.5 Phosphate glass fibres (PGFs) re-enforced scaffold manufacture

#### 2.5.1 Production and characterization of PGFs

PGFs with the following formula 50P2O5.30CaO.20Na2O mol.% [41] and a diameter of 20 ± 2 μm were continuously drawn at 30 ms−1 directly from a glass melt using an in-house melt drawing facility. The composition of the glass fibres was examined through Energy Dispersive X-ray (EDX) analysis (Oxford Instruments INCA, UK, connected with JEOL 6490 LV SEM) with an accelerating voltage of 15 kV at a working distance of 10 mm, spot size of 50, and at 150× magnification. The glass fibres were mounted on double-sided carbon tape and coated with carbon (Quorum coater). The composition analysis was run through map and point analysis on large and small areas at several points (minimum 8) to quantify the amount of P, Na, Ca, and O present. The EDS data, targeted and actual formulation are all summarized in supplementary materials provided with the manuscript.

#### 2.5.2 Creation of PGFs’ scaffold

**Fig. 2:**
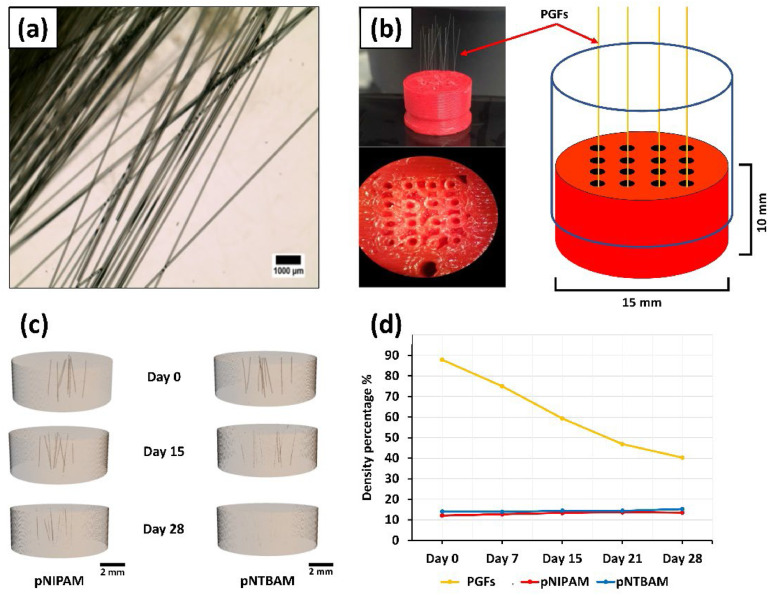
PGFs alignment in scaffold moulds and their degradation pattern. (a) Microscopic image for bio-glass fibres; (b) PGFs loaded onto 3D printed mould; (c) μCT scanned images of hydrogel embedded PGFs showing PGF mass (brown colour) threaded within hydrogels (transparent) captured with time (0, 15, and 28 days). Images scale bar at 2 mm; (d) Density reduction (%) of FGFs tracked across 28 days and compared to hydrogel density.

A 3D printed mould model was created that comprised a cylindrical polymeric base with channel holes to position PGFs vertically (Fig. 2). The mould was designed on Autodesk Autocad 2012 software and printed on a Makerbot 3D printer. Non-biodegradable acrylonitrile butadiene styrene (ABS) was used for printing. Mould dimensions were 15 mm diameter x 10 mm thickness. The mould's holes, to host the PGFs, were arranged in a 4 × 4 configuration with a diameter of 1 mm (Fig. 2b). The polymers were prepared and cast over the mould, as above, to generate a gradient scaffold with incorporated PGF. Scaffolds were then sealed and stored at room temperature till use.

### 2.6 PGF degradation

The degradation pattern for PGF mass was tracked with an X-ray Scano micro-CT40 set at 55 kvp/ 71 μA. Scanned samples were analysed to verify the variable densities between hydrogel and PGF mass per material volume. The scanned samples were analysed to verify the fibre mass density per hydrogel by showing the dense object mass (PGF fibres) in the transparent low-density hydrogel mass. Each component was analysed according to specific density thresholds revealing the PGF mass at the higher threshold (134–1000) versus the hydrogel mass at the lower threshold (0-134). 3D images were constructed enabling PGF fibre degradation tracking at 0, 7, 15, 21, and 28 days after incubation with PBS at 37 °C.

### 2.7 Biological assessment of gradient and PGFs enforced scaffolds

#### 2.7.1 Cell culture

Primary human osteoblasts (hOBs) and primary human chondrocytes (hCHs), both are cryopreserved primary cells supplied by Promo Cell®. These were isolated from the femoral trabecular bone tissue (knee and hip) and from normal human articular cartilage (knee and hip) respectively. The supplier protocol for culturing was followed for both types of cells with passage 2 was utilized for experimental work. The selected primary cells were intended to explore the osteogenic versus chondrogenic differentiation potential presented by these cells upon scaffold seeding.

Scaffolds were washed with PBS for 48 h at 37 °C to remove any monomeric and chemical residues. The PBS solution was changed every 4–8 h. Scaffolds were sterilised by immersing in 99 % ethanol solution for 20–30 min. Samples were then washed again with sterile PBS for 24 hours at 37 °C. A final washing step was performed by soaking the samples in media for 1-2 hours before seeding hydrogels with cells. Scaffold samples were sliced vertically, and cells seeded upon the exposed internal surface. A seeding density of 105 cells/cm^2^ was applied.

#### 2.7.2 Glycosaminoglycans (GAGs) assessment

Alcian Blue staining test was used for the detection of mucopolysaccharides and glycoproteins (sulphated and non-sulphated), which constitute an active cartilage matrix component. A 1% w/v Alcian Blue solution was prepared by dissolving 0.5 g of alcian blue 8GX (Sigma) in 50 mL of 3 % v/v acetic acid solution at room temperature with pH subsequently adjusted to 1.5. Samples were covered with alcian blue staining solution, sealed, and incubated for 24 h at room temperature with gentle agitation. Thereafter, the stain solution was aspirated from the sample which was then washed 5X with dH2O over 24 h. Microscopic observation and imaging of samples was carried out via Lieca dissection and EVOS XL core bright field microscopes.

The presence of sulphated glycosaminoglycans (GAGs), another major cartilage matrix component, was established using Dimethyl methylene blue (DMMB) colorimetric reagent (1,9-Dimethyl-methylene Blue zinc chloride double salt (Sigma)). For DMMB analysis a working solution was prepared by mixing 0.008 g DMMB reagent, 1.52 g glycine, and 1.185 g sodium chloride with 500 mL d H2O before stirring for 3–4 h at room temperature in the dark, the pH adjusted to 3, and then stored at room temperature in the dark until use. DMMB was solubilized first with 5 mL absolute ethanol before mixing. Samples were removed from culture media, washed 3X with PBS, freeze dried, and minced with scalpels. Samples were then digested with 1 mL papain digestion buffer, sealed and incubated at 60 °C overnight. In a 96-well culture plate, sample lysates and standards (both in triplicate) were added at 50 µL each. A 250 µL DMMB working solution aliquot was then added and the absorption immediately taken using Synergy II BioTek plate reader plate reader at 525 nm. Chondroitin sulphate was used as a standard solution by dissolving 2.5 mg chondroitin sulphate in 50 mL d H2O at room temperature. A serial dilution was then prepared from the stock solution to reflect the standard readings.

#### 2.7.3 Calcium mineral assessment

The identification of calcium minerals was performed with alizarin red S pigment (Sigma) and calcium colorimetric assay kit (Sigma). Alizarin red stain induces chelation with calcium minerals to produce a red alizarin-calcium complex indicating evidence of calcium mineral association [52]. Alizarin red solution (1% w/v) was prepared and adjusted to pH 4.2, sealed, and stored at room temperature. Samples were removed from culture, washed 3X with PBS, and fixed with 10 % v/v formaldehyde. Each sample was soaked with alizarin staining solution, ensuring that the sample was adequately covered. Samples were left on a rotary shaker for 30 min at room temperature. The dye was then removed and samples washed with dH2O for 24 h with water being changed 5 times. A final PBS wash step was carried out for 15 min at room temperature. Microscopic images were taken with an EVOS XL core brightfield microscope.

Quantification of calcium minerals upon scaffolds was assisted with colorimetric calcium assay. The assay is based on the ability of O-cresolphthalein reagent to combine with calcium ions present in the sample to induce colour change [53]. Samples were first removed from culture media then fixed with 10 % v/v formaldehyde for 30 min at room temperature. Fixed samples were then washed 3 times with dH2O, followed by freeze-drying. A 0.5 M diluted HCl solution was used to break the calcium ion bonding to polymer surfaces, releasing calcium into solution. A 24-well plate was used as a base container for the hydrogel samples. Then, samples were incubated for 24 h in 0.5 mL of HCl extraction solution, sealed with para-film, and agitated overnight. Next day, the seal was removed, and the solution collected from each sample well. Using a 96-well plate, 50 µL of each collected sample and control solutions was added followed by 90 µL of chromogen reagent to prompt the complex formation. To clearly illustrate the colour differentiation in solution, 60 µL of calcium buffer solution was added with gentle mixing. Reaction plates were incubated at room temperature and protected from light for 5-10 min before having absorbance measured on Synergy II BioTek plate reader at 575 nm.

#### 2.7.4 Immunostaining

Primary antibodies for collagens type I (Anti-Collagen I antibody (ab34710, Abcam)), II (Anti-Collagen II antibody (ab34712, Abcam)), and X (Anti-Collagen X antibody (ab58632, Abcam)) were used. These were visualised with secondary antibodies (Goat Anti-Rabbit IgG H&L (Abcam)) labelled with TRITC or FITC. Scaffolds were fixed with 10 % v/v formaldehyde for 30 min at room temperature before being blocked with 5% w/v bovine serum albumin (BSA) in PBS for 3 h at 4 °C. This was followed by incubation with primary antibody solution overnight at 4 °C. The primary solution was prepared by mixing individual primary antibody with 5% w/v BSA in PBS at a 1:200 ratio. The primary antibody solution was then aspirated and samples washed four times for 5–10 min with a 1% w/v BSA in PBS solution. Samples were then incubated with Goat Anti-Rabbit IgG secondary antibody (FITC or TRITC) in 5 % BSA in PBS solution at 1:200 ratio. Samples were incubated at 4 °C for 4 h in the dark before washing with 1 % BSA in PBS 5 times (5–10 min each), followed by 2 washes in PBS. Nuclear staining was performed by incubating samples with DAPI stain for 30 min at room temperature, then washing 3X with PBS. To locate any fluorescent indication of protein expression, hydrogels were observed under Olympus U-TBI90 laser fluorescent confocal microscope. Settings for confocal imaging, including laser intensity, brightness, and contrast, were adjusted at the same levels for all samples.

#### 2.7.5 ELISA testing

Scaffold samples were examined for collagens I, II, and annexin A2 using Sandwich enzyme-linked immunosorbent assay (ELISA). The assayed markers should indicate the way the cells are reacting to their 3D environment and weather they are in line with their function. Annexin A2, however, should reflect the cells response in laying out minerals to the surrounding matrix. Samples were washed 3X with PBS before freeze drying and digesting with papain digestion buffer overnight. Sample lysates were then collected in eppendorf tubes and frozen at −80 °C until needed. Sample lysates were assessed for total protein content using Bicinchoninic acid (D8284 Sigma) protein assay. A 7.5 % bovine serum albumin solution was used as a standard to verify the total protein amounts of samples. Samples were normalised to the lowest protein content by diluting with the original digestion buffer. The assay procedure was carried out at room temperature with Nunc® immunoassay 96 microplates. All the assay kits (Human Pro-Collagen I alpha 1 DuoSet ELISA, Human Pro-Collagen II DuoSet ELISA, and Human Total Annexin A2 DuoSet IC ELISA) were supplied by R&D systems. Assay procedure was followed according to manufacturer's recommendations.

A 1% w/v BSA in PBS solution was used as a blocking buffer and 0.05 % v/v Tween® 20 in PBS used as washing buffer. A 2,2′-azino-bis (3-ethylbenzothiazoline-6-sulfonic acid) (supplied by Sigma) substrate was used to complete the reaction. Microplate wells were coated with 100 µL /well of capture antibody solution. Plates were sealed and incubated at room temperature overnight before capture antibody solution was removed, and plates washed X3 with 400 µL/well of wash buffer. This was followed by blocking with 300 µL/well of blocking buffer for 2 h at room temperature before washing as previously described. Samples and standards were then applied at 100 µL/well in diluent buffer, plates sealed and incubated at room temperature for 2 hours. Plates were then washed again as before, and detection antibodies added, sealed and incubated at room temperature for 2 h. Following on from washing, samples were incubated with streptavidin reagent for 20 min at room temperature. Plates were then washed as previous, and a final incubation with substrate reagent for 20 min at room temperature. Stop solution was then added at 50 µL /well and absorbance measured immediately at 450 nm wavelength.

### 2.8 Statistical analysis

All data were collected and analysed with Microsoft Excel to calculate the mean, standard deviation and resulting graphs. Results obtained were compared using one and two-way ANOVA with Tukey's multiple comparisons test. Statistics were analysed using Origin Pro 8, the level of significance was set at *P* ≤ 0.05.

## 3 Results

### 3.1 Gradient hydrogel architectural properties

#### 3.1.1 General scaffold appearance

Following casting, the resultant hydrogel scaffolds (measuring 10 mm width × 15 mm thickness) revealed a gradual visual change in characteristics along the gradient axis (Fig. 1a). The observant change was reflected in a transition from transparency, pNIPAM, into an opaque white, pNTBAM. (Fig. 1b).

#### 3.1.2 FTIR

The major chemical discrepancies between the two polymers were identified at the lower energy level of spectral chart. The fingerprint band region at 1200 cm⁻1 displayed distinctive spectral peaks for isopropyl and t‑butyl compounds. Basically, two bands were recognized at 1131 cm-1 and 1171 cm-1 respectively for pNIPAM polymer, whereas a single wide band appeared for pNTBAM at 1224 cm-1. The rest of the spectra demonstrated the main functional groups at the higher energy level for both polymers’ structures. A basic identification was for the CH3, NH, and C = O stretch spectral bands.

Sequential FTIR scans revealed a progressive change of spectral peaks from pNIPAM at 1131 cm⁻1 and 1171 cm⁻1 to pNTBAM at 1224 cm⁻1 likely arising from C = O stretching changes due to the additional methyl group. The interface region spectra revealed a gradual spectral peak switch moving from a pNIPAM to a pNTBAM profile which may indicate the integration between polymers chains (Fig. 1c).

#### 3.1.3 SEM

Images from SEM revealed variable architecture across scaffolds length featuring three distinct regions of different porosity. The top and bottom regions were indicative of pNTBAM and pNIPAM highlighting their distinctive porous structure. The interface region displayed its own distinctive pore shape and size, predominated by flake-like polymer aggregates with a larger pore diameter. Pore diameter measurements indicated a larger mean pore size for pNIPAM (54.46±10.3 µm) when compared to pNTBAM (16.5 ± 4.5 µm) (*P* ≤ 0.05). Pore size in the mixed region was significantly larger (118.34±24.5 µm) than both pNIPAM and pNTBAM regions (*P* ≤ 0.05) (Fig. 1d).

### 3.2 Phosphate glass fibre (PGF) mass evaluation

#### 3.2.1 Micro computed tomography (µCT)

The PGFs (Fig. 2a) were manually inserted within scaffold's mould holes (Fig. 2b). PGFs were detected within cast hydrogels via µCT. Image analysis revealed PGF presence and distribution within the hydrogel composite that decreased over 28 days of incubation in PBS at 37 °C (Fig. 2c). Density percentages plotted against time revealed PGFs mass reduction compared to a less dense hydrogel mass, where PGF mass ratio was evaluated per total sample volume (Fig. 2d).

### 3.3 Biological assessment of gradient scaffold

#### 3.3.1 Chondrogenic differentiation

**Fig. 3:**
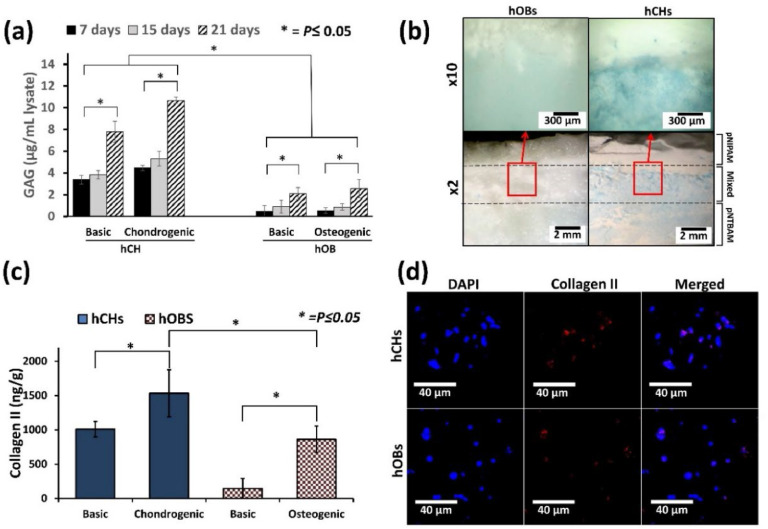
Chondrogenic differentiation in pNIPAM-pNTBAM gradient scaffolds. (a) GAG (μg/mL lysate) in scaffold samples seeded with hOBs and hCHs and observed at 7, 15, and 21 days in either basic (control), chondrogenic, or osteogenic culture media; (b) Alcian blue stained gradient hydrogel sections seeded with hOBs and hCHs, images illustrate gradient scaffolds captured at X2 and X10. The interface region (open red square) is magnified in the X10 image. Scale bar = 2 mm for X2 and 300 μm for X10 images; (c) ELISA quantification of collagen II (ng/g) after 21 days of hOBs and hCHs. Asterisks indicate significance at *P* ≤ 0.05; (d) Confocal images of collagen II-labelled gradient scaffolds seeded with either hOBs or hCHs. DAPI, Collagen II, and merged images are provided for both hOB and hCH seeded scaffolds. Scale bar = 40 μm. Asterisks indicate significant levels at *P* ≤ 0.05. Error bars indicate ±SD, *n* = 3.

DMMB assay revealed higher GAGs associated with gradient scaffolds seeded with hCHs (Fig. 3a). GAG levels were significantly higher when hCHs were cultured in chondrogenic media than in basic media and when compared to hOB seeded gradient scaffolds in osteogenic media. Monitoring GAGs over time indicated a progressive increase for all gradient scaffolds, irrespective of cell or media used. Consistent with above Alcian blue indicated little to no staining in hOB-seeded gradient scaffolds. In contrast to hOBs, hCHs-seeded gradient scaffolds displayed robust Alcian blue staining on the pNTBAM region of the scaffold and limited staining within the pNIPAM zone (Fig. 3b).

ELISA testing for collagen II showed that gradient hydrogels seeded with hCHs displayed elevated collagen II after 21 days when cultured in chondrogenic media vs. basic media (*P* ≤ 0.05) (Fig. 3c). Similarly, gradient hydrogels seeded with hOBs in osteogenic media displayed significant elevation of collagen II at (*P* ≤ 0.05 vs. basic) after 21 days. Overall, hCHs in chondrogenic media displayed significantly elevated collagen II production when compared to all other samples. Confocal image analysis confirmed expression of collagen II (red fluorescent) with hCHs and hOBs samples (Fig. 3d).

#### 3.3.2 Mineralization and osteogenic differentiation

**Fig. 4:**
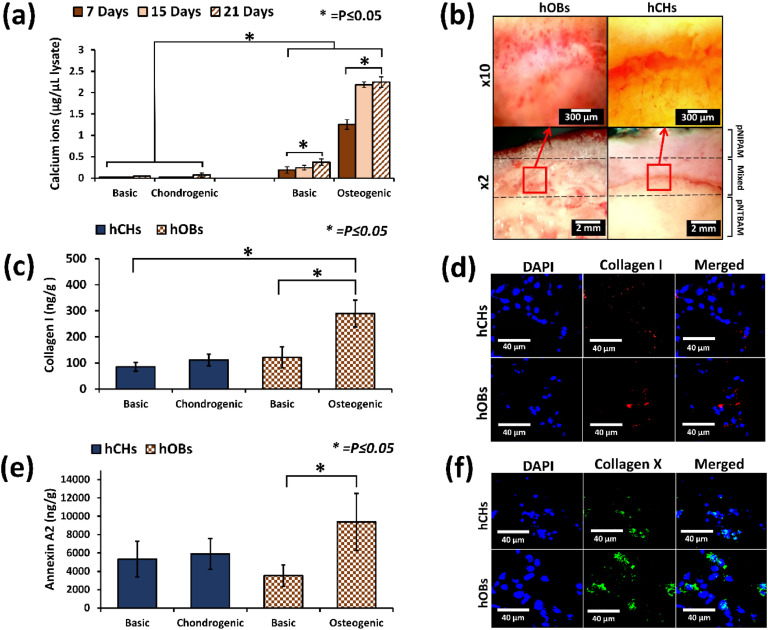
Osteogenic differentiation of hOB and hCH in pNIPAM-pNTBAM gradient scaffolds. (a) Calcium ions (μg /μL lysate) compared between hOB and hCH scaffolds at days 7, 15, and 21; (b) Alizarin-stained gradient hydrogel sections seeded with either hOBs or hCHs. Images are whole sample (X2) and (X10) for the interface region (open red square). Scale bar = 2 mm for X2 and 300 μm for X10 images; (c) ELISA quantification of collagen I (ng/g) on gradient hydrogels seeded with hOBs and hCHs; (d) Confocal images of Collagen I immunolabelled gradient samples seeded with hOBs and hCHs; (e) Annexin A2 (ng/g) in gradient hydrogels seeded with hOBs and hCHs; (f) Confocal images of Collagen X immunolabelled gradient samples seeded with hOBs and hCHs. Confocal images showing cells with DAPI, collagen, and annexin A2 immunolabelling including merged images at x40. Asterisks indicate significant levels at *P* ≤ 0.05. Error bars indicate ±SD, *n* = 3.

Calcium ion quantification indicated increased levels in hOB-seeded gradient scaffolds cultured in osteogenic media when compared to basic media cultured scaffolds (*P* ≤ 0.05) (Fig. 4a). Little or no calcium ions were detected in hCH-seeded gradient scaffolds irrespective of chondrogenic or basic media use (*P* ≤ 0.05). Consistent with above hOB-seeded gradient scaffolds displayed strong staining that was abundant throughout the scaffold surface (Fig. 4b). Gradient samples with hCHs showed less alizarin red staining across the sample surface but did display evidence of calcium minerals at the interface region (Fig. 4b).

Collagen I detection via ELISA indicated increased levels in hOB-seeded gradient scaffolds cultured in osteogenic media when compared to basic (*P* ≤ 0.05) (Fig. 4c). Collagen I level in hCH-seeded gradient scaffolds were detectable at similar levels irrespective of which media formulation was applied, but significantly lower than that observed with hOBs in osteogenic media. Consistent with these observations collagen I expression was detectable by immunofluorescence in both hOB- and hCH-seeded gradient scaffolds (Fig. 4d).

Gradient scaffolds seeded with hOBs displayed elevated annexin A2 levels when cultured in osteogenic (9396.28 ng/g) compared to basic media (3523.6 ng/g) (*P* ≤ 0.05) (Fig. 4e). hCH-seeded gradient scaffolds displayed similar levels irrespective of media used (5892.25 ng/g chondrogenic and 5331 ng/g basic). Collagen X, a marker for hypertrophic cartilage, expression was detected in gradient scaffolds seeded with either cell type (Fig. 4f). Consistent with collagen I and Annexin A2 levels and Alazarin Red staining the hOB-seeded gradient scaffold displayed apparently higher levels of collagen X labelling by immunofluorescence. Evidence of collagen X expression was also noted in hCH-seeded gradient samples.

### 3.4 PGF embedded scaffold assessment

#### 3.4.1 Calcium minerals and GAGs

**Fig. 5:**
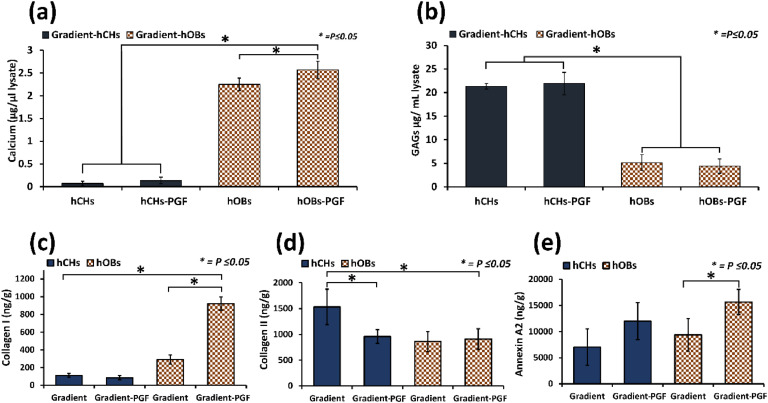
Osteogenic and chondrogenic cell differentiation markers in PGF vs non-PGF gradient scaffolds. (a) Calcium ion comparison at day 21 of culture between PGF and non-PGF gradient samples seeded with hOB or hCH; (b) GAGs in PGF and non-PGF gradient samples seeded with hOB or hCH at day 21 of culture; (c, d, e) ELISA quantification of Collagens I, II, and Annexin A2, respectively, at day 21 of culture in PGF vs. non-PGF gradient samples. Asterisks indicate significant levels at *P* ≤ 0.05. Error bars indicate ±SD, *n* = 3.

Calcium mineral was significantly elevated in hOB-seeded PGF gradient scaffolds when compared to non-PGF gradient scaffolds (2.57 µg/µl vs. 2.24 µg/µl, *P* ≤ 0.05) after 21 days. hCH-seeded PGF gradient scaffolds revealed no significant increase of calcium minerals when compared to non-PGF scaffolds (Fig. 5a).

The incorporation of PGFs provided no measurable impact on GAG levels vs non-PGF gradient scaffolds. GAG production was equivalent for both when seeded with hCHs and significantly higher than hOB-seeded gradient scaffolds, again irrespective of PGF incorporation (*P* ≤ 0.05) (Fig. 5b).

#### 3.4.2 ELISA detection of proteins for PGF scaffolds

We sought to determine the impact of PGFs on matrix-associated protein secretion within gradient scaffolds. Collagen I was significantly increased in hOB seeded PGF gradient scaffolds when compared to non-PGF scaffolds both cultured in osteogenic media (*P* ≤ 0.05). hCH-seeded gradient scaffolds displayed little collagen I irrespective of PGF incorporation with chondrogenic media (*P* > 0.05) (Fig. 5c). Collagen II levels were significantly lower (*P* ≤ 0.05) for gradient-PGF scaffolds seeded with hCHs than in non-PGF samples. With hOB-seeded scaffolds levels of collagen II were comparable to hCH-seeded PGF scaffolds and not significantly altered between hOB PGF and non- PGF scaffolds (Fig. 5d).

Annexin A2 was significantly elevated (*P* ≤ 0.05) in gradient-PGF samples seeded with hOB (15,660±2444 ng/g) compared to the non-PGF scaffolds (9396.2 ± 3107.5 ng/g). Although not significant (*P* > 0.05), PGF-scaffolds’ samples seeded with hCH revealed evidence of enhanced mineralization potential (11,997±3547 ng/g) relevant to the non-PGF samples (7014.6 ± 3504.4 ng/g) (Fig. 5e).

## 4 Discussion

The cartilage-bone interface is an integrated region offering a gradual transition between bone and cartilage tissues [54]. In the present work, pNIPAM and pNTBAM hydrogels were assembled to produce a 3D multi-regional scaffold to evaluate their future potential as a tool in gradient tissue development. Previous studies have explored the utility of multi-layer scaffolds in regeneration of complex tissue constructs such as the osteochondral region [55]. These studies had researched the possibility of combining different materials such as collagen I and hydroxyapatite and declared the potential of this bi-layered construct to support bone and cartilage regeneration [[56], [57], [58]]. Some success has been reported in guided multiple tissue regeneration in utilising materials of variable characteristics including collagen I, hydroxyapatite, alginate, and chitosan [59]. A recent focus has been on creation of integrated material scaffolds incorporating functional gradients to better reflect natural tissues [[60], [61], [62]]. pNIPAM and pNTBAM are two thermos responsive monomers that have been widely studied for their potential applications in drug delivery, tissue engineering, and bioimaging [63]. These polymers are attractive due to their ability to undergo a reversible phase transition from hydrophilic to hydrophobic in response to temperature changes. This property allows for the controlled release of drugs or the selective targeting of specific tissues [64]. Further combination of these polymers enhanced the biocompatibility and cell adhesion to variable limit which could an interesting aspect when conducting the current study [35,40].

The gradient hydrogel we describe presents clear integration with no evident junctional margins to differentiate between the two polymers (Fig. 1b). Several techniques have been used to fabricate materials into gradient constructs with variations as per materials characteristics and the tissue targeted for regeneration [61,[65], [66], [67]]. Matyjaszewski, et al. described the eligibility of atom transfer radical polymerization (ATRP) for gradient polymers production making use of the polymerization process to blend materials of variable properties [68]. This method of joining the current materials allows for the development of integrated regions between the two polymers. The timing of addition of each polymer layer ensured that the polymerisation process initiated in the first layer would build progressive polymer chains with sequential layers. Accordingly, the interface region between the layers was integrated by infiltrating polymer chains forming intact bonding regions. Thereby creating a single construct featuring three architectural regions with an avoidance of the delamination seen when combining different materials in traditional multilayer scaffolds [69].

The active functional groups distinguished in pNIPAM and pNTBAM hydrogels, as determined according to FTIR spectral analysis, may elucidate aspects of their materials’ impact upon reaction with the biological system. The presence of functional groups such as C = O and NH- contributes to heightened hydrophilic tendencies in the polymer surface, potentially fostering active osteoblastic differentiation activity [70,71]. This aligns with established research indicating a positive correlation between hydrophilicity and enhanced material's biocompatibility [72]. The presence of CH3- groups contributes to some hydrophobic characteristics. The relatively augmented presentation of CH3- groups in pNTBAM potentially accounts for the subtle hydrophobic nature observed in this polymer in comparison to pNIPAM. The more relative expression of CH3- groups by pNTBAM and the interplay with the more hydrophilic pNIPAM may enhance cell adhesion to materials’ surfaces which was noticed in a combined pNIPAM/pNTBAM polymer film model [35,73]. The identified CH3- band deformation according to FTIR revealed at around 1200 cm-1 in pNTBAM and around 1100 cm-1 in pNIPAM corresponds to each polymer specific character or fingerprint region [74]. Tracking these regions assisted in monitoring the exchange in a mixed region of the co-pNIPAM/pNTBAM polymers.

The development of a multiregional scaffold was illustrated by a combination of FTIR characterisation and SEM imaging. The FTIR spectra indicated a gradual transition from pNTBAM to pNIPAM with a distinct blended spectra at the interface zone. This was obvious when the fingerprint bands for each polymer infiltrates each other with gradual vanishing of the pNIPAM two bands while evolving of the pNTBAM single band (Fig. 1c). This may emphasize the development of an interface region between the two polymers that could possibly features different characteristics. This was obvious when observing the SEM images across scaffold's length, verifying multi-architectural regions from the pNIPAM to pNTBAM reinforced by pore size. Pore size was smaller for pNTBAM than with pNIPAM while the interface zone pores were largest of all reflecting a discontinuous gradient porosity. Thus, although the current scaffold may present three distinct regions with no structural gradient, a gradient polymeric transformation was observed between the two sides of the scaffold. Wettability variations between the two polymers impacted the process of their production with specific, individual, solvent requirements. Such a difference between polymers’ solubility requirement will affect the final architectural outcome when both monomers’ solutions come in contact with each other during gradient scaffold production. In this case the presence of an alcohol component, i.e., methanol from pNTBAM solvent mixture, affects the swelling properties of pNIPAM polymer leading to formation of a macroporous hydrogel at the interface region [75]. Alternate solvents are described as impacting the architectural properties of polymers. For example, the use of an N-Methyl-2-pyrrolidone solvent in preparing a PLGA/nHA scaffold resulted in a 16 % decrease in porosity vs. dioxane solvent [76]. This concurs with our porosity observations in the interface region of the pNIPAM-pNTBAM scaffold (Fig. 1d). According to the natural osteochondral interface architecture, the sub-chondral bone region has a large porous structure that is followed by the sub-chondral bone plate, which is a more dense bony region, before moving to the calcified cartilaginous region [[77], [78], [79]]. The current design on the pNIPAM scaffold side plus the interface region gave rise to a larger porous structure which hosted higher rate of mineralization when tested with cellular behavior thus mimicking the sub-chondral bone plate [80,81]. Moving towards the pNTBAM side of the scaffold could represent the calcified cartilage side as it has more capability of hosting chondrogenesis and mineralization by cells.

The current cell work involves seeding directly to the surface of the scaffold without encapsulation. The cytoxicity of monomeric units required the avoidance of direct encapsulation and repeated washing of the produced scaffold to ensure complete removal of any remaining monomers. The polymers’ cytotoxicity and cell survival, utilizing primary cells, has been tested and proved in our previous manuscript for characterizing pNIPAM and pNTBAM polymers, separately [34]. We observed via histology, specific behaviors of cells upon scaffold's variable architectural regions. GAG production was strongest for hCHs at the pNTBAM side and interrupted at the interface with pNIPAM. Calcium mineral association was enhanced across a wide area of scaffold regions including the interface, mostly with hOBs. This strongly indicates a role for the resultant scaffold's architecture in guiding cell behaviour. Consistent with our observations, differentiation of human mesenchymal stem cells (hMSCs) on a gradient scaffolds produced from poly(ethylene oxide therephtalate)/poly(butylene therephtalate) (PEOT/PBT) and of poly(ε -caprolactone) (PCL) with porous structure resulted in an increased chondrogenic behaviour and GAG production in smaller pore regions [66]. Conversely, hMSCs displayed increased osteogenic differentiation and mineralization when associated with a larger porous architecture. Further, an exploration of variable porosities of poly-l-lactide-co-trimethylene carbonate scaffolds on cartilaginous matrix production an enhanced activity was associated with smaller pore regions [82]. Mineralisation was also evident in association with hCHs at the interface region (Fig. 4b). We hypothesize that the mixed polymer interface region, with large porous architecture, provided a supportive background for the engagement of our hCHs in mineralisation activity [22]. It has been reported elsewhere that a large porous architecture is an excellent promoter for enhanced osteogenic and mineralization behaviour of cells [80]. In this case, the mixed polymer region of the current scaffold may support a dense mineral region owing to the larger porous structure. The enhanced collagen X expression observed at the interface region by hCHs was in further support of this assumption. In a study by Korpayev, et al., a multi-layered gradient scaffold with nano hydroxyapatite was assessed for osteochondral tissue regeneration. The engagement of MC3T3 and ATDC5 chondrocytes in an osteoblastic activity was indicated by significant expression of collagen X. The study refers to the impact of the background scaffold construct and composition on guiding cell functionality to regenerate the osteochondral interface consistent with the current study [83]. Annexin A2 is detected in matrix vesicles produced by these cells in calcified cartilage zone and in sub-chondral bone[84]. ELISA identification of annexin A2 provides further evidence for the mineralization process. Annexin A2 and collagen X can be utilised as indicators for calcium mineralization of the extracellular matrix by hypertrophic chondrocytes and osteoblasts[84]. Reviewing other markers, collagens I and II localisation was consistent with hOBs and hCHs, respectively, on gradient scaffolds (Fig. 4, Fig. 5) suggesting that these cells were providing evidence of functionality relevant to their type.

Scaffold utility in osteoarthritic defect correction was explored by design of in situ injectable alginate/polyvinyl alcohol blends that incorporated alternate layered chondroitin and hydroxyapatite nanoparticles [85]. The resultant compositional variation affected crosslinking of the polymer network resulting in a pore size gradient where mineralized activity was dominant at the larger pore area of the scaffold with more chondrogenic activity by chondrocytes at the smaller pores side of the scaffold. Although our work compares well with others in the presentation of a single scaffold presenting a structured range of pore size across its length corresponding with chondrogenic differentiation towards smaller pores [82,86], the material reported here provides larger pore size at the pNIPAM-pNTBAM interface region enabling greater control of spatial cell response across the gradient.

Osteochondral scaffold design must include consideration of tissue vascularization and mass transfer, through inclusion of design features including porous architecture [10,49,87]. Embedded PGFs were proposed for the current thermos responsive scaffold to enable cell migration and support vascular infiltration from the sub-chondral layer [82]. Further, PGF degradation is considered as a potent stimulator for osteogenic activity and bone matrix formation [28,88]. In the present scaffold, reduction of fibre mass was ongoing at day 28 where remnants remained visible. The impact of incorporating PGFs in a PVA hydrogel on chondrocyte performance was explored [89]. This determined that PGF mass volume reduction plateaued at day 28 and that the degradation was associated with enhanced chondrocyte proliferation and performance.

The presence of PGFs enhanced calcium mineralisation in samples seeded with hOBs [90]. PGF gradient scaffolds seeded with hOBs displayed significantly higher annexin A2 levels when compared to non-PGF samples (both incubated in osteogenic media). This implies that PGFs and their degradation, encouraged osteogenesis. A gradient scaffold comprised of polycaprolactone (PCL) with gradients of chondroitin sulphate and sol-gel bioactive glass revealed the engagement of chondrocytes in a mineralized activity [91]. This is consistent with our current results demonstrating mineralization behaviour when PGF mass is incorporated in a hydrogel. In assessing samples with hCHs, no significant difference was seen with PGF samples compared to non-PGF. However, ELISA measurements identify evidence of elevated annexin A2 levels for PGF samples with hCHs. These findings may be interesting when realizing that hCH scaffolds’ samples showed significant reduction with collagen II in non-PGF samples (Fig. 5d). This is a possible indication of engaged chondrocytes with mineralization and, probably, a hypertrophic potential. These observations are consistent with previous reports that PGFs enhanced osteoblasts’ and chondrocytes’ mineralisation activity [28,[92], [93], [94]]. Chondrocyte association with minerals (as per increased annexin A2 levels) is a key attribute of the hypertrophic chondrocyte that forms the calcified cartilage matrix [78]. While no data is provided to demonstrate transformation into hypertrophic chondrocytes, we have demonstrated evidence of a transitional change towards mineralization where the expression of annexin A2 may reflect a future trend of these cells to produce matrix vesicles and mineralize the surrounding matrix. In this prospective, the current scaffold design along with PGFs may presents a successful model of supporting osteogenic to chondrogenic differentiation with enhanced matrix mineralisation. This may further aid the regeneration of the complex osteochondral interface featuring a mineralized gradient that mimics the calcified cartilage and subchondral bone. In the context of previous studies featuring multilayer scaffolds [22,24,83], our model presents a fortified construct aided by the polymers’ chains bonding scaffolds regions. In view of the recent advances in autologous chondrocyte implantation (ACI), a hydrogel based ACI provided enhancement as the hydrogel construct provided guidance for cells [95]. The engagement of the collagen biphasic scaffold into ACI, helped to accelerate tissue healing with better results in term of cartilage tissue type [95,96]. However, this was applicable for the superior cartilage layers and questions remain on its effectiveness in calcified and subchondral bone layers. Our current design presents a model for full thickness defect by involvement of the sub-chondral bone and calcified cartilage layers which could improve the effectiveness of hydrogel-based ACI. The inclusion of certain green nano materials such as silk, chitosan, or carbon nanoparticles [[97], [98], [99], [100]], may also represent additional improvements for the current design in the context of cartilage and bone tissue regeneration.

The current study may have some limitations that we think it is important to be consider when interpreting the findings. Although the current polymers’ stiffness were explored in our previous manuscript [34], testing mechanical strength of the current scaffold would aid more comprehension on scaffold's stiffness and biological cell responses. Also, the current scaffold presented visually intact structure that can easily be handled experimentally. However, addressing scaffold's stability and solubility in various environmental conditions and pH would have proven the strength of the current scaffold design. Despite these limitations, the findings of the current study provide valuable insights on the impact of variable chemistry and architecture on biological cell responses and mineralization. These findings may spot the light on how the current combinations may serve the regeneration of complex tissue construct such as the osteochondral interface.

## 5 Conclusions

Osteochrondral defects give rise to impairment of movement and pain in joints. Strategies to repair and regenerate this tissue are difficult due to the different types of materials needed to support the cellular architecture and structural characteristics of the ECM to provide tissue function. Multi-materials suffer from delamination with gradient materials presenting a range in surface chemical and physical pore characteristics offering the potential for this use. The closely-related polymers, pNIPAM and pNTBAM, were suitable for creation of a multi-regional scaffold with differing architectural zones. Each zone presented specific pore diameter with smaller pore size being at pNTBAM side (16.5 µm) compared to larger pores by pNIPAM side (54.5 µm) but significantly larger pore size at the mixed middle zone (118 µm). The mixed scaffold's zone displayed a capacity for modulation of both osteogenic and chondrogenic differentiation in a cell specific manner. The chondrogenic potential was enhanced by the pNTBAM side of scaffold compared to pNIPAM side. The different chemistry and porosity between the two polymers allowed for variations in cell responses while moving across the scaffold. The larger pores in the mixed interface hosted more mineral activity by cells while the smaller pores of pNTBAM addressed for more chondrogenic differentiation potential. PGFs into scaffolds’ construct encountered for an enhanced mineral and osteogenic activity with hOBs with some evidence reported in hCHs samples evidenced by elevated collagens I, X, and annexin A2 with reduction of collagen II in PGFs scaffolds. This has potential to increase production of a mineralized interface by engaging chondrocytes to produce a calcified matrix; a key component of the osteochondral interface. The development of a mixed polymer, PGFs-incorporated, gradient scaffold has been demonstrated for exploration as an osteochondral injury therapeutic.

## Funding

The authors acknowledged the funding support from the Higher Committee for Education Development (HCED) Iraq.

## Acknowledgments

The authors would like to thank Dr. Joshua Price, Dr. Tina Dale, Dr Hamza Abu Owida and Dr Marwan Merkhan for help and advice with experimental lab work. Also, special thanks to Dr. Ann Canning for her help in the 3D printing design to support the current project.

## Ethical approval

No human or animal experiments took place within this study.

## CRediT authorship contribution statement

**Zaid M. Younus:** Writing – original draft, Software, Methodology, Investigation, Funding acquisition, Data curation, Conceptualization. **Ifty Ahmed:** Writing – review & editing, Validation, Resources. **Paul Roach:** Writing – review & editing, Validation, Supervision, Methodology, Investigation, Conceptualization. **Nicholas R. Forsyth:** Writing – review & editing, Validation, Supervision, Methodology, Investigation, Data curation, Conceptualization.

## Declaration of competing interest

The authors declare the following financial interests/personal relationships which may be considered as potential competing interests: Zaid M. Younus reports financial support was provided by The Higher Committee for Education Development in Iraq. If there are other authors, they declare that they have no known competing financial interests or personal relationships that could have appeared to influence the work reported in this paper.

## Data Availability

Data will be made available on request.

## Appendix Supplementary materials

Image, application 1
