# Supplementary material for: A phosphate glass reinforced composite acrylamide gradient scaffold for osteochondral interface regeneration
Source: Biomater Biosyst. 2024 Jul 26;15:100099. doi: 10.1016/j.bbiosy.2024.100099 (PMC11364006; doi:10.1016/j.bbiosy.2024.100099)
Supplement: Supplementary file 1 [file mmc1.docx]

**Supplementary Information**

**Manuscript title:**

**A Phosphate Glass Reinforced Composite Acrylamide Gradient Scaffold for Osteochondral Interface Regeneration**

**Table 1.** Essential kits and materials utilized in the current study detailed with their percentage concentrations, catalogue number, and suppliers.

| Material | Catalogue No. | Supplier |
| --- | --- | --- |
| 1,9-Dimethyl-methylene Blue zinc chloride double salt | 341088 | Sigma-Aldrich |
| Alcian blue 8GX | A3157 | Sigma Aldrich |
| Alizarin Red S | A5533 | Sigma-Aldrich |
| Ammonium persulfate, reagent grade 98% | 215589 | Sigma-Aldrich |
| Anti-Collagen I antibody | ab34710 | Abcam |
| Anti-Collagen II antibody | ab34712 | Abcam |
| Anti-Collagen X antibody | ab58632 | Abcam |
| Bicinchoninic acid | B9643 | Sigma Aldrich |
| Bovine Serum Albumin solution 7.5 % | A8412 | Sigma Aldrich |
| Calcium Colorimetric Assay Kit | MAK022 | Sigma-Aldrich |
| DAPI (4′,6-diamidino-2-phenylindole) | D9542 | Sigma Aldrich |
| Goat Anti-Rabbit IgG H&L (FITC) | ab6717 | Abcam |
| Goat Anti-Rabbit IgG H&L (TRITC) | ab6718 | Abcam |
| Human chondrocytes (HCH) cryopreserved | C-12710 | Promo Cell |
| Human osteoblasts (HOB) cryopreserved | C-12720 | Promo Cell |
| Human Pro-Collagen I alpha 1 DuoSet ELISA | DY6220-05 | R & D Systems |
| Human Pro-Collagen II DuoSet ELISA | DY7589-05 | R & D Systems |
| Human TGF-beta 3 (E.coli) | 100-36E | PeproTech |
| Human Total Annexin A2 DuoSet IC ELISA | DYC3928-5 | R & D Systems |
| N,N,N′,N′-Tetramethylethylenediamine, reagent plus 99% | T22500 | Sigma-Aldrich |
| N,N′-Methylenebisacrylamide | M7279 | Sigma-Aldrich |
| N-Isopropylacrylamide | 415324 | Sigma-Aldrich |
| N-tert-Butylacrylamide 97% | 411779 | Sigma-Aldrich |
| Papain from papaya latex | P4762 | Sigma-Aldrich |

**Table 2**. Composition of PGFs (confirmed according to EDX analysis) displaying the molar percentages for each component, elemental weight percentage (wt%), and EDS spectrum.

|  | Mol. % | | |
| --- | --- | --- | --- |
|  | P2O5 | CaO | Na2O |
| Target formulation | 50 | 30 | 20 |
| Actual formulation | 50.96 | 29.99 | 19.05 |
| Elemental wt% | | | |
| O | Na | P | Ca |
| 48.22 | 8.58 | 31.31 | 11.88 |
| 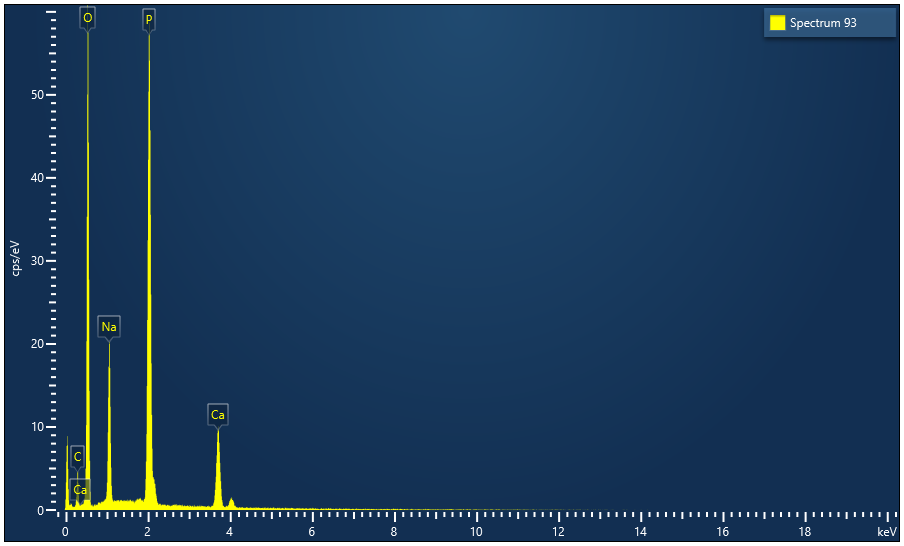EDS spectrum | | | |
